# Measuring the visual environment of children and young people at risk of myopia: a scoping review

**DOI:** 10.1007/s00417-024-06719-z

**Published:** 2025-01-22

**Authors:** Annegret H. Dahlmann-Noor, Desta Bokre, Marina Khazova, Luke L. A. Price

**Affiliations:** 1 University College London Institute of Ophthalmology, 11-43 Bath Street, London, EC1V 9EL UK; 2 NIHR Moorfields Biomedical Research Centre, London, UK; 3 Moorfields Eye Hospital NHS Foundation Trust, London, UK; 4 Radiation, Chemicals, Climate and Environmental Hazards Directorate, UK Health Security Agency, Didcot, UK

**Keywords:** Myopia, Daylight, Near work, Electronic devices, Child, Adolescent

## Abstract

**Purpose:**

Myopia (short-sightedness) is an emerging WHO priority eye disease. Rise in prevalence and severity are driven by changes in lifestyle and environment of children and young people (CYP), including less time spent in bright daylight and more time spent on near-vision activities. We aimed to systematically map the literature describing direct, objective measurements of the visual environment of CYP.

**Methods:**

We conducted searches in Ovid Medline 1946, Ovid Embase and The Cochrane Central Register of Controlled Trials in November 2024. We included primary research written in English on environmental/behavioural factors and myopia onset/progression in CYP 3–18 years. Two reviewers independently screened titles/abstracts/full texts.

**Results:**

We included 34 articles: 21 explored the association of indoor and/or outdoor light exposure and myopia and included light measurements, two near-vision activities, four both light and near-work, four time outdoors without illuminance measurements, and three light exposure based on meteorological data. Most measurements were carried out at the level of individual children, rather than the surrounding environment alone.

**Conclusion:**

Despite limitations in measurement techniques, there is evidence that reduced illuminance, less time spent in bright light and increased daily duration/sustained episodes of near-vision activities and reduced working distance are associated with increased myopia prevalence/progression.

**Supplementary Information:**

The online version contains supplementary material available at 10.1007/s00417-024-06719-z.

## Abstract (highlights)

****Key messages**:**

*****What is known***:**

- Rise in prevalence and severity of childhood myopia are driven by changes in lifestyle and environment of children and young people (CYP), including less time spent in bright daylight and more time spent on near-vision activities.
- A large variety of tools and devices are used to measure these factors.

*****What is new***:**

- Many studies include lifestyle questionnaires rather than objective measurements.
- Most measurements were carried out at the level of individual children, rather than the surrounding environment alone.
- Standardisation of approaches and use of objective measurement techniques for cohorts of children would allow harmonisation of research findings.

**Supplementary Information:**

The online version contains supplementary material available at 10.1007/s00417-024-06719-z.

## Introduction

### Rationale

Changes in lifestyle and environment of children and young people (CYP) are the driving force behind the recent sharp increase in the prevalence and severity of myopia (short-sightedness) [1]. Prevalence is around 47.2% in young adults in Europe [2], and over 90% in East Asian countries; by 2050, half the world population may have myopia [3]. Earlier onset and faster progression expose higher numbers of adults to the risk of suffering permanent loss of vision and blindness from myopia-related complications [4, 5]. Specifically, the prevalence of myopic retinopathy increases from 0.42% in people with myopia less than 5 diopters to 25.3% in those with myopia of 5 diopters or more [6]. This increased risk of myopia-associated complications is commonly expressed as Odds Ratio (OR): the OR of myopic macular degeneration, compared with people without myopia, is 2.2 for those with myopia of −1.0 to −2.99D, 9.7 for those with myopia of −3.0 to −4.99D, 40.6 for −5.0 to −6.99D of myopia, 126.8 for −7.0 to −8.99D, and 348.6 for myopia of −9.00D and greater [6]. For retinal detachment, the OR is 3.1 for those with myopia of −0.75 for −2.75D, 9.0 for −3.0D to −5.75D, 21.5 for −6 to −8.75D, 44.2 for −9 to 14.75D, and 88.2 for −15D and higher [6]. For myopic optic neuropathy, which is often grouped under glaucomatous optic nerve damage, the OR is 1.6 for those with myopia up to −3.00D, and 2.5 for those with −3.00D or higher degrees of myopia [6]. Lastly, people with myopia have a higher risk of developing cataract, with an OR of 2.1 for −1 to −3.5D, 3.1 for −3.5 to −6D, and 5.5 for myopia greater than −6D [6].

The WHO has designated refractive errors as a priority eye disease [7]. The main factors driving increasing myopia prevalence and its faster progression are a reduction in exposure to high-intensity outdoor light, as children spend more time indoors, and probably an increase in daily duration and sustained use of near-vision activities, as educational demands have increased, particularly in urban settings, and as many CYP use screens for leisure activities and socialising [8–11]. Systematic reviews and meta-analyses have confirmed that increasing time outdoors and reducing near-work time are effective in reducing myopia incidence in school-age children, but had a limited effect on myopia progression [12]. They also pointed out that the quality of the evidence was often limited due to recall bias, as researchers used parent- and child-completed questionnaires to estimate these factors [10, 12]. Further risk factors for myopia development may be suboptimal indoor lighting at school and at home, and near-working distance of less than 30 cm [11]. Currently unknown is whether reduced exposures to specific wavelengths of light, i.e. the blue/violet and/or red regions of the spectrum, contribute to the rise in myopia prevalence. Technology is increasingly available to measure both children’s exposure to light and their near-work habits, offering more detailed data exploring the impact of these factors on myopia and the effectiveness of light- and near-work-based interventions [11, 13, 14].

### Objectives

With the recent and ongoing rapid developments of measurement techniques and evidence base about new interventions, a scoping review is an appropriate initial step to comprehensively summarise the collective evidence regarding the impact of measured illuminance in the child’s immediate environment and at eye-level, and that of measured near-activities on myopia onset and progression. Specific objectives were:

1. To describe which measurement technologies have been used to assess the visual environment of children (parameters related to light exposure and near-work), both at individual level and for clusters of children, for example schools, and
2. To enable researchers to identify gaps in knowledge that could be addressed by established and novel measurement techniques and by combining individual and cluster-level measurements.

## Methods

### Protocol and registration

We conducted the review in accordance with the Preferred Reporting Items for Systematic Reviews and Meta-analysis Protocols/Extension for Scoping reviews (PRISMA-ScR) [15]. The protocol was registered on Open Science Framework on July 15, 2023.

### Eligibility criteria / information sources

We included studies reporting investigations with CYP from age 3 to 18 years and focussed on environmental and behavioural observations and interventions to delay the onset of myopia or slow its progression, such as changes to habitual light exposure (indoors and outdoors), or changes to duration or distance used during near-focus activities. We included peer-reviewed journal articles published until the day of the search (12 Nov 2024), written in English. We included primary research articles with any study design, and secondary data analyses. Included studies had to report light- or near-vision activity measurements of verifiable and representative exposure conditions (such as school timetable interventions, not parental questionnaires) and at least one of the three following outcomes: myopia incidence/prevalence, axial length, spherical equivalent refraction. We excluded articles in other languages, animal and cellular/tissue studies, optical (myopia management glasses and contact lenses) or pharmacological interventions, and interventions involving devices for targeted ocular delivery of light of particular wavelengths to an individual. We excluded articles which explored interventions delivered within a hospital, clinic or practice setting; measurements of outcomes such axial length and spherical equivalent in these settings were permitted. We excluded meta-analyses, systematic and scoping reviews, commentaries, editorials and grey literature.

### Search

We carried out searches in Ovid Medline 1946, Ovid Embase 1947 and The Cochrane Central Register of Controlled Trials. The key terms used were “myopia”, “sunlight, or daylight activities”, and “electronic devices”. We used Medical Subject Headings (MeSH) and free text terms with all alternative synonyms and derivatives to capture all relevant studies. We also used Boolean operators “OR”, “AND” to combine search lines and applied age limits to retrieve studies on children up to age of 18 years (Supplementary Tables 1–3).

### Selection of sources of evidence

We exported search results from databases to an EndNote library to remove duplicate records. We transferred references from Endnote to Covidence Systematic Review Software (Veritas Health Innovation, Melbourne, Australia) for further de-duplication and screening. To ensure consistency among reviewers during title and abstract screening, we developed an abstract screening tool. For piloting, two reviewers, A-DN and LP, independently screened the same selection of 20 titles and abstracts from the search, discussed the results, and amended the abstract screening tool. LP and A-DN then screened all titles and abstracts, discussed and resolved disagreements within Covidence, with reviewer MK as mediator when required. We included publications for full-text review where the abstract review had indicated “unsure”. Two reviewers, A-DN and LP, then independently scrutinised the full texts for inclusion using a custom-designed inclusion/exclusion criteria list within Covidence. We discussed and resolved disagreements, with MK as mediator.

### Data charting process and data items

We developed a data extraction tool within Covidence, which included: publication identifying information (first author and author contact details, year, title, country), study aims, design, setting (including indoor/outdoor), start/end date, funding sources, conflicts of interest, target population, inclusion/exclusion criteria, recruitment methods, number of participants and duration of study participation, duration of daily observations/measurements, population demographics (mean age, gender, proportion of young people with myopia), intervention(s) if any, parameters studied, technology used to measure light and near-work parameters, summary statistics of intervention parameters and of myopia-related outcomes at baseline and follow-up timepoints. We tabulated the characteristics of included studies (number, geographic distribution, populations, study designs). We then analysed the content and summarised the findings of included studies according to interventions (if any) and outcomes. We did not carry out a quality assessment of included studies, because the objective of this scoping review was to provide a map and overview of the research conducted to date.

In line with guidance for scoping reviews [15], we did not carry out a critical appraisal of individual sources.

## Results

### Selection of sources of evidence

**Fig. 1:**
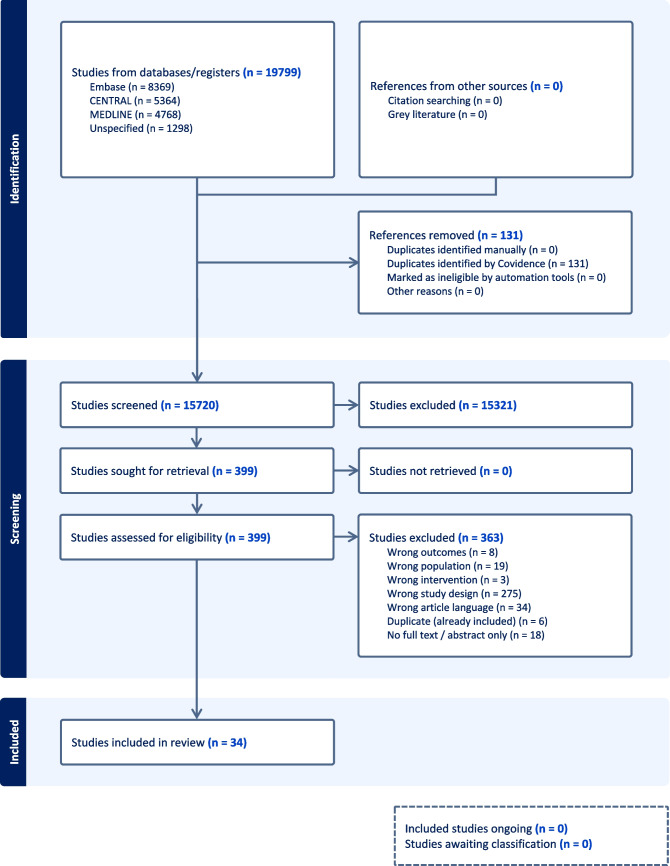
PRISMA flowchart of scoping review process

We retrieved 4,768 references from Ovid Medline 1946, 8,369 references from Embase 1947 and 5,364 from The Cochrane Central Register of Controlled Trials. We updated our searches across the 3 databases at later stage to retrieve further 1,298 references. Altogether, we exported 19,799 references to EndNote and removed 3,948 duplicate records to reduce the total number of references to 15,851. Then, we exported 15,851 references from EndNote to Covidence and removed further 131 duplicate records, resulting in 15,720 records. Two independent reviewers screened abstracts and titles, eliminating 15,321 studies. Of 399 full-texts reviewed, 34 were included in the scoping review (Fig. 1).

### Characteristics of sources of evidence

The most commonly used study designs in the included studies were cross-sectional (*n* = 13), cohort (*n* = 7) and non-randomised experimental designs (*n* = 4, Table 1). Most studies enrolled children under 13 years of age (*n* = 23) (Table 1). Asia/Pacific was the predominant region of origin of included studies (*n* = 26, Table 1). Funding sources were government/national/local research bodies; no commercial funders were identified. Publication dates ranged from 2003 to 2024. Data collections in the included studies started between October 1999 and November 2021 and ended between February 2010 and March 2023.

**Table 1:** Characteristics of included studies

Classification	Number	Classification	Number
Study design (*n* = 34)		Region (*n* = 34)	
Cross sectional study	13	Asia Pacific	26
Cohort study	7	Europe	3
Non-randomised experimental study	4	Americas	2
Randomised controlled trial	4	Middle East/Africa	3
Other *	6	**Country (*n* = 34)**	
Population (*n* = 34)		China	15
Children under 13 years	23	Australia	3
Age 13 and over	0	Israel	3
Children/young people of all ages	11	Korea	1
		Taiwan	2
		Singapore	2
		USA	2
		Hong Kong	1
		India	1
		Japan	1
		Czech Republic	1
		Denmark	1
		Netherlands	1

*Other study designs: Cluster randomisation of schools (*n* = 2), others *n* = 1 each: Behaviour/exposure/methods study, Cross-sectional with elements of case-control, Non-randomised interventional, Secondary analysis of cohort study data

Most commonly used enrolment sites were schools and clinics (Table 2). The number of participants enrolled ranged from 36 to 435,996. In most studies (*n* = 25), the settings where measurements were collected included home, school and leisure, less frequently at school only (*n* = 8); one study acquired measurements at home only. Most studies acquired measurements during both outdoor and indoor time (*n* = 20), whilst others focussed on one setting (indoors only *n* = 5, outdoors only *n* = 5).

**Table 2:** Enrolment sites, settings, place and type of measurements acquired

	Number of studies
Enrolment site (*n* = 35 studies*)	
Through school	19
Clinic patients or their families	5
Through other research studies	7
Volunteers responding to advertisements in clinics, university, and on social media	3
Not stated	1
Setting during measurements (*n* = 34)	
Home and school and leisure	25
School only	8
Home only	1
Measurements indoors/outdoors (*n* = 34)	
Both indoor and outdoor **, ***	20
Indoor only	5
Prescribed time outdoors only	5
Daylight hours only ****	4
Parameters measured (*n* = 34)	
Light parameters only **, ***	21
Near-work parameters only	2
Both light and near-work	4
Prescribed time outdoors only	4
Daylight hours only ****	3

^*^Enthoven 2021 used two enrolment strategies; **He et al. 2022 [16] and Wu et al. 2018 [17] prescribed time outdoors and reported light measurements. ***Torii et al. 2017 [18] reported UV transmittance of eyewear ****Leng et al. 2021 [19] and Ma et al. 2024 [20] reported average annual sunshine hours from meteorological data

The number of studies acquiring measurements at the level of the individual child was greater than for those acquiring measurements at room or other surrounding levels, both for light-parameters (19 vs 4 studies, Table 3) and for near-work parameters (all at individual level, Table 4). Studies exploring light parameters were more frequent than those studying near-work parameters (28 studies including 5 which used meteorological data, rather than measurements acquired during the study, vs 6 near-work studies).

**Table 3:** Light measurement characteristics and devices

Individual or environment	Light measurement technology	Light parameter measured	Study ID
Individual	HOBO Pendant Pendant temp/light Part # UA-002–64	Intensity only	Dharani et al. 2012 [21], Wu et al. 2018 [17], Li et al. 2022 [22]
	Actiwatch 2	Intensity only	Read et al. 2014 [23], Read et al. 2015 [24], Landis et al. 2018 [25]
	Actiwatch Spectrum (model not specified, Pro or Plus in photo)	Intensity only	Gordon-Shaag et al. 2021 [26]
	Actiwatch Spectrum Plus	Intensity only	Mirhajianmoghadam et al. 2021 [27], Shneor et al. 2023 [28]
	Actiwatch Spectrum Plus	Intensity and spectral composition	Ostrin et al. 2018 [29]
	Clouclip	Intensity only	Wen et al. 2020 [30]
	Clouclip	Intensity and duration	Li et al. 2020 [31], Zhang et al. 2023 [32]
	FitSight wrist-worn watch (patent WO2015152818A1)	Intensity only	Li et al. 2021 [33]
	Akeso sensor	Intensity only	Fan et al. 2022 [34]
	Smart watch ‘Mumu’ equipped with a light sensor, accelerometer and GPS receiver	Illuminance, UV irradiance	He et al. 2022 [16], Chen et al. 2024 [35]
	Eye-wear transmittance measured with UV-2600 spectrophotometer	Spectral composition only	Torii et al. 2017 [18]
	MyLyt Tracker	Intensity and duration	Dakhal et al. 2024 [36]
Environment	Luxmeter Lutron LX-101A, Lutron Electronics Co Inc	[Horizontal illuminance inferred] in lux in the centre of the nursery room in April 2017 between 10 am and 12 noon	Cohen et al. 2022 [37]
	HOBO Pendant Pendant temp/light Part # UA-002–64 [not worn; used for classroom daylight factor assessment]	Intensity only	Suh et al. 2022 [38]
	Lux meter TES 1330	Average illuminance (intensity) and illuminance uniformity (minimum to average ratio) for desks and blackboards from evening measurements of electric light	Hua et al. 2015 [39]
	spectral measurement instrument (Model OSP-350S, Hangzhou Ouhong INTELLIGENT Technology Co. Ltd, http://www.ohomesmart.com/)	Intensity, spectral composition	Cai et al. 2024 [40]
Meteorological data		local daylight hours using astronomical table (include intensity, timing, duration, solar elevation; hours of darkness), regardless of actual exposure	Cui et al. 2013 [41]
	annual sunshine duration	city-level 10-year climatic data	Leng et al. 2021 [19]
	meteorological data; for analysis: season only taken into account (summer, winter)	local daylight hours	Hecova et al. 2023 [42]
	China Meteorological Data Sharing Service (http://Data.cma.cn/) data from 2018–2020	average monthly sunshine duration for 103 areas, defined as duration of direct sunlight from sunrise to sunset (> 120 W/m^2^), unaffected by shading (clouds, fog), measured in hours	Ma et al. 2024 [20]
	NASA/NOAA’s Visible Infrared Imaging Radiometer Suite (VIIRS) Day/Night Band (DNB) low-light imaging data	Artificial Light at Night: Radiance, nW/cm^2^/sr (at satellite) which is proportional to reflected irradiance (at surface level)	Liu et al. 2024 [43]

Where studies include terms “lux”, “(light) intensity” and “(light) exposure” to refer to illuminance, the approach here is to use the same term in quotations. Some devices reporting illuminance measurements at the level of the child may not be generally accurate, e.g. due to instrument limitations spectral, directional or dynamic sensitivity characteristics, even for a calibrated device [44]

**Table 4:** Near-work measurement characteristics and devices

Individual or environment	Technology used to measure near work	Near work parameter measured	Study ID
Individual	Clouclip	Distance and duration, Li also calculation of visual behaviour index	Wen et al. 2020 [30], Li et al. 2020 [31, 45], Zhang et al. 2023 [32]
	MyopiaApp and face-screen distance measure (camera) on smartphone	Distance and duration	Enthoven et al. 2021 [45]
	Akeso	Distance and duration	Fan et al. 2022 [34]
	Kinect-for-Windows	Dioptric volume (the total amount of net defocus)	Choi et al. 2020 [46]

Most studies collected data for up to one year, most enrolled boys and girls in approximately equal proportions (where reported), and most included children with and without myopia (Table 5).

**Table 5:** Participant demographics and measurement characteristics

	Number of studies
Gender distribution (*n* = 34)	
Boys/Girls in roughly equal proportion	15
Boys only	1
Not stated	18
With/without myopia (*n* = 29)	
With and without myopia	21
With myopia only	4
Without myopia only	1
Not stated	3
Duration of study participation for individual participants (*n* = 34)	
1 week or less	8
More than 1 week, up to a month	6
More than 1 month, up to one year	12
More than one year, up to 3 years	6
More than 3 years	2
Duration of daily measurements (*n* = 34)	
From 1 to less than 6 h	1
From 6 to less than 14 h	5
More than 14 h, up to "continuous"	11
"Daylight hours"	1
Not applicable	16

### Results of individual sources of evidence

**Fig. 2:**
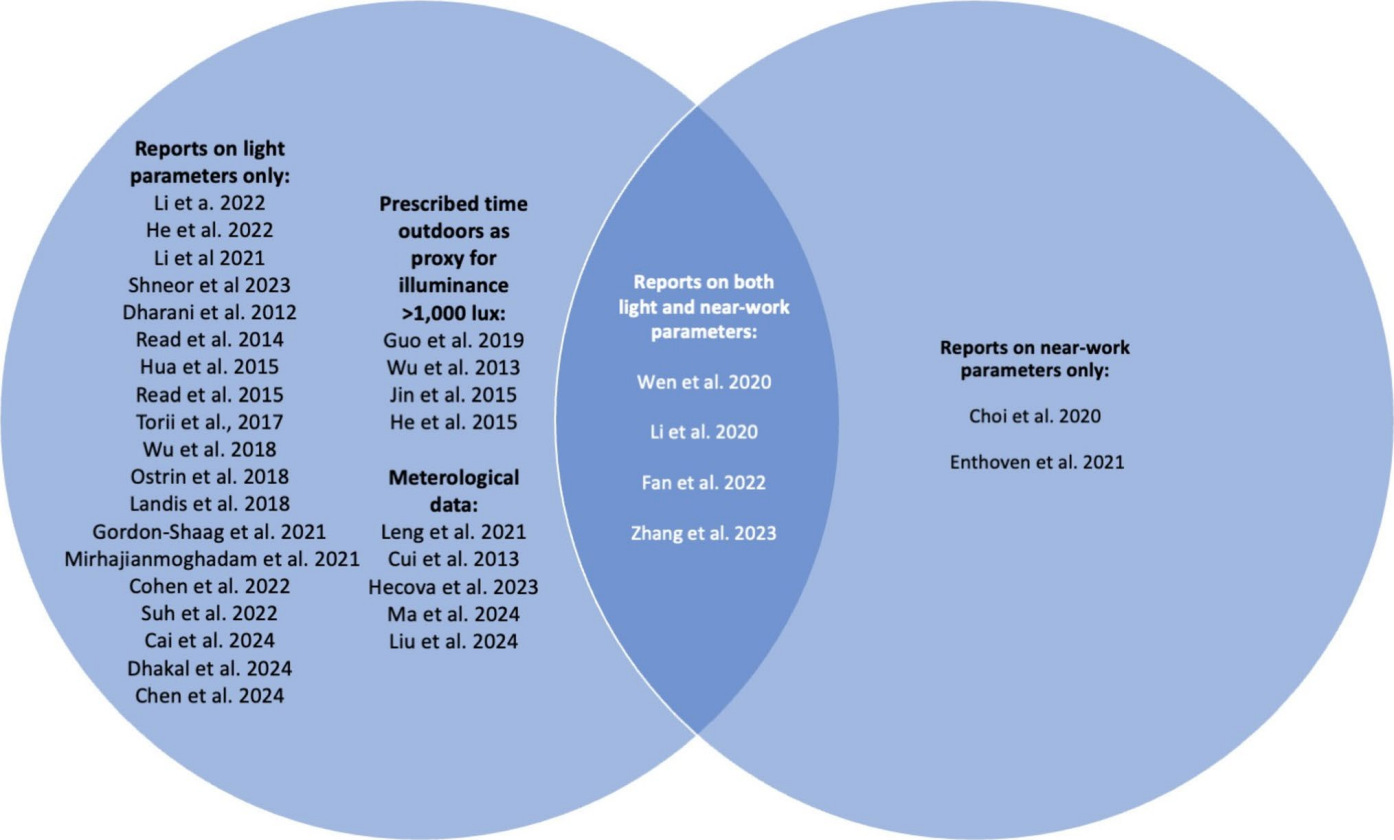
Venn diagram of included studies

Nineteen studies explored light parameters only and included light measurements, two near-vision parameters only, three both light and near-vision parameters, four described “time outdoors” as a proxy for exposure to bright light, verifiable by school timetables, and five estimated light exposure based on meteorological data, including daylight hours (Fig. 2).

#### Reports on light parameters only

##### Measures

A variety of wearable and handheld Illuminance meters have been used both to measure the light conditions at the level of the individual child and to measure classroom/home/environmental lighting. Results were often presented with non-standard terminology and units, and we have re-expressed this in standard form as far as practicable.

**Measurements of indoor and outdoor environmental illuminance**

Studies in this category measured room illuminance at defined locations in classrooms, such as desks and the board, but measurement direction did not necessarily coincide with the child’s direction of viewing.

As children spend a large portion of their daytime hours in educational settings, lighting levels in schools and nurseries may be highly relevant to myopia onset and progression. A study in Israel measured horizontal illuminance in the centre of nursery rooms at 27 nurseries, as well as outside, using a Lutron LX-101A illuminance meter (Lutron Electronics Co Inc, USA) [37]. Nurseries were categorised based on mean “light intensity”, into low, medium and high intensity groups. Mean illuminance ranged from 359 lx in the low-intensity to 490 lx in the medium-intensity and 671 lx in the high-intensity group (Table 4) [37].

In schools in Korea, measurements acquired with a HOBO UA-002–64 Pendant Waterproof Temperature & Light Logger (“HOBO Pendant”, Onset Computer Corporation, USA) showed a daylight factor ranging from 0.51% to 13.35% [38].

An interventional study using cluster-randomisation of schools in China explored the impact of improved lighting on myopia parameters [39]. Intervention schools received suspension-mounted grille luminaires with fluorescent tubes, hung from the ceiling parallel to the window in two rows, and a separate blackboard lamp fixture [39]. Desk illuminance increased from 74 lx to over 300 lx, and blackboard illuminance from 71 lx to over 500 lx [39]. Another recent interventional study designed classrooms with artificial light to mimic natural light (“artificial natural light”, (Guangdong Cosio Lighting Co. ltd, http://www.cosiolighting.com/) [40]. The light source switches of classrooms were changed and connected to the network, and the use of lamps could be monitored in real-time. A spectral measurement instrument (Model OSP-350S, Hangzhou Ouhong INTELLIGENT technology Co. Ltd, http://www.ohomesmart.com/) measured the spectral and lighting parameters of the classroom light source. The spectrogram of light source in the intervention classrooms had a peak at around 530 nm, corresponding to green colour; that of the control classroom, 580 nm, yellow colour [40]. There was no change in long-wavelength end of the spectrum [40].

Meteorological data have also been used to assess children’s potential daylight exposure at a collective level. In Denmark, characterised by long days in summer and long nights in winter, children followed for 6 months starting at different times of year were grouped into categories based on mean “accumulated hours of daylight” from an astronomical table. The highest category had a mean of 2782 h, and the lowest, 1681 h [41]. In similar studies, annual sunshine duration was also obtained from meteorological institutions in China [19, 20], and daily duration of sunshine during the observation period in a study in the Czech Republic [42]. A different type of data was used in a study in China: artificial light at night were sourced from NASA/NOAA’s Visible Infrared Imaging Radiometer Suite (VIIRS) Day/Night Band (DNB) low-light imaging data [43]. This measured radiance in nW/cm^2^/sr at satellite, which is proportional to reflected irradiance at surface level from a Visible Infrared radiometer (waveband of measure not reported) [43].

**Light exposure measurements at the level of the individual child**

Studies in this category used devices worn as pendants or on the wrist; none used spectacle-mounted devices that would measure illuminance at eye-level.

Children wearing a HOBO Pendant revealed that children in Singapore spend a mean time of around 7 h/week at illuminances of > 1000 lx during termtime, and 9.8 h/week during holidays [21]. Both during termtime and during holidays, children spend more time per day at illuminances of > 1000 lx on weekends than on weekdays, though the difference was not as marked during the holidays as during termtime. Mean illuminance levels children were exposed to were around 700 lx during school-time, and 950 lx during school holidays [21]. Correlation with parental diaries of children’s activities was at best fair; the authors concluded that recall bias may affect diary completion, whilst the lack of an agreed cut-off illuminance level to define “outdoor” and “indoor” time made interpretation of findings difficult [21].

HOBO Pendants were also used within a cluster-randomised controlled trial of schools in China, which tested the impact of spending 11 h or more per week outdoors [17]. Children wore the pendant for 7 days; 49.79% of children in the intervention group and 22.73% in the control group spent 11 h or more per week at illuminances of > 1000 lx.

Lastly, HOBO Pendants were worn for 3 days by children whose parents received text messages (SMS) twice daily for a year, reminding them to take their children outdoors [22]. During the intervention period, children in the SMS group spent greater time outdoors and experienced greater light exposure (presumably mean daily illuminance) than those in the control group, though only on the weekends (0.34 vs 0.18 h and 46 vs 28 lx) [22]. One explanation for the reported low mean daily illuminance at weekends might be inclusion of non-compliant data from devices not being worn, or obscured by clothing.

Different models of the Actiwatch (Koninklijke Philips N.V., NL), a wrist-worn activity (actigraph) and light logging device, have been used in several studies exploring the association of light exposure and myopia. A study in Australia used the Actiwatch 2, observing that children with myopia had a mean daily illuminance of 915 lx, whilst their peers without myopia were exposed to a mean of 1272 lx [23]. They also spent less time in light > 1000 lx (91 min/day versus 127 min/day) [23]. A longitudinal analysis showed that the mean daily illuminance encountered by children with myopia was 805 lx, compared with 999 lx in children without myopia [24].

A re-analysis of data collected by children wearing an Actiwatch 2 in the Role of Outdoor Activity in Myopia (ROAM) study in Australia to explore the effect of spending time in dim light showed that children with myopia spent more time in dim/mesopic lighting (defined as illuminances of 1 lx to 30 lx) than children without myopia (weekdays: 5.56 h/day vs 5.16 h/day, weekends 6.50 h/day vs 5.75 h/day) [25]. Children with myopia also spent less time in outdoor bright/photopic light (defined as > 1000 lx: 1.35 h/day vs 1.85 h/day on weekdays and 1.27 h/day vs 1.93 h/day on weekends) [25].

Using an Actiwatch Spectrum Plus, one study in the USA measured both “light intensity” and “spectral composition” [29]. Children spent a mean of 110.5 min/day outdoors during the summer, more than in the other seasons: 94.2 min/day in spring and 72.2 min/day in the fall. The highest light dose (“mean daily light exposure”) was measured in the summer. Exposure to red/green/blue components highly correlated with exposure to white light [29].

A study in Israel also using an Actiwatch Spectrum Plus compared light exposure of boys attending different types of educational settings [26]. The Actiwatch Spectrum Plus reports “white light” in the units of illuminance, i.e. lux, which is sometimes reported as a proxy for illuminance [47]. Time outdoors ranged from 1.77 h/day to 2.38 h/day on weekdays, with lower mean daily illuminance at schools with higher educational demands, and from 1.85 h/day to 2.52 h/day on weekends [26].

Another study in Israel used the Actiwatch Spectrum Plus to compare time outdoors and “white light exposure” in ultra-orthodox, religious and secular boys [28]. The authors reported that ultra-Orthodox boys spent significantly less time outdoors (presumably daily mean data) than religious and secular boys on weekdays (ultra-Orthodox: 0.99 ± 0.53 h; religious: 1.19 ± 0.55 h; secular 1.36 ± 0.61 h, *P* = 0.02) and overall (weekdays and Shabbat combined: ultra-Orthodox: 0.85 ± 0.4 h; religious: 1.24 ± 0.55 h; secular: 1.37 ± 0.6 h, *P* = 0.002). Ultra-orthodox boys were also exposed to significantly less average “white light” than secular (*P* = 0.01) and religious boys (*P* = 0.04) overall (ultra-Orthodox: 298 ± 114 lx; religious: 382 ± 160 lx; secular: 403 ± 156 lx) [28].

A US-based study using an Actiwatch Spectrum Plus reported that “mean [white] light exposure” of children with myopia was 183.6 lx, compared with 279.5 lx in children without myopia [27]. During the COVID-19 pandemic, children spent less time outdoors [27].

A different wrist-worn watch, FitSight, was used in a study in Singapore and showed a significant difference between diary-reported mean (± SD) time outdoors of 100 ± 93 min/day and measured time outdoors (≥ 1000 lx: 37 ± 19 min/day) [33]. Average light levels children were exposed to were 458 ± 228 lx, with peak light exposure at mid-day [33].

An observational study using a wrist-worn smartwatch, Mumu, measured the light exposure of children with myopia, and analysed duration of time spent outdoors and sunlight intensity, categorising “exposure patterns” [35]. Mumu was also used within a cluster randomised trial to measure illuminance and UV irradiance [16]. Children prescribed an additional 40 or 80 min of time outdoors per day showed similar outdoor time and “light intensity” (40 min: 127 ± 30 min/day and 3557 ± 970 lx/min; 80 min: 127 ± 26 min/day and 3662 ± 803 lx/min) but significantly more than the control group (106 ± 27 min/day and 2984 ± 806 lx/min) [16].

Another wearable device, MyLyt, clipped to clothing, was used in a study in India to compare light exposure in children with and without myopia [36]. Children with myopia had a median illuminance exposure of 382 (IQR 247–594) lux/day, and those without myopia, 491 (IQR 289–735) lux/day; this difference was not statistically significant [36].

A study exploring specific parts of the spectrum of wavelengths of light used eyewear reducing transmission of wavelengths below 400 nm, referred to as violet light [18]. Blocking of certain wavelengths was confirmed by spectroscopy.

##### Associations of illuminance with and impact on myopia onset and progression

**Studies exploring room/spatial illuminance**

In the study in Israel that measured illuminance at nursery schools (mean age of children 4.87 (SD 0.33) years), children attending the nurseries within the low-intensity group had a mean noncycloplegic spherical equivalent of + 0.56D, those in the medium-intensity group, + 0.73D, and those in the high-intensity group, + 0.89D. In the low-intensity nurseries, 42.1% of children had an autorefractor reading of zero or less (myopia), compared with 19.3% in the high-intensity group [37].

The daylight factor in school classrooms correlates with myopia parameters: in the school with the lowest vs highest daylight factor (DF), children had a higher spherical equivalent at baseline (−0.09D vs −0.41D) and six months later (−0.34D vs −0.64D). Axial length also differed both at baseline (22.83 mm low DF vs 22.96 mm high DF) and follow-up (22.98 mm vs 23.15 mm). The rate of progression was not significantly different between the lowest and highest DF schools in the overall cohort, but in those children with AL of less than 22.7 mm at baseline, the rate of elongation over 6 months was significantly higher in those attending the low DF school [38].

Increasing desk and blackboard lighting significantly reduced the prevalence of new-myopia-onset (4% vs 10%), myopic shift (−0.25D vs −0.47D), and axial length increase in children without myopia (0.13 mm vs 0.18 mm) and those with myopia (0.20 mm vs 0.27 mm) over 12 months in a study population with a mean age of around 10 years in China [39]. Artificial Natural Lighting was associated with a modest reduction in incident (new-onset myopia): 164/774 vs 207/784 students over 3 years (21 vs 26%) [40].

In the study exploring duration of daylight exposure in Denmark, mean axial elongation was significantly lower in children in the highest-exposure group than in the lower-exposure group (0.12 mm vs 0.19 mm over 6 months), and spherical equivalent progression was also lower (−0.26D vs −0.32D) [41]. Similarly, both studies in China correlating daylight exposure with myopia prevalence observed that longer annual sunshine duration was associated with lower myopia prevalence (OR 0.721, 95% CI 0.593 to 0.877, [19]; for each 1-unit increment in sunshine duration, there was a 0.4% decreased risk of myopia (OR = 0.996; 95% CI 0.995–0.998; *P* < 0.001, [20]). The study exploring levels of artificial light at night (ALAN) in China observed that children with myopia lived in areas with higher ALAN exposure than those without myopia (median 14.44 vs 6.95 nW/cm^2^/sr, CI 3.88–26.56 vs 1.21–21.74) [43]. Comparing progression in different seasons, the study in the Czech Republic observed that axial elongation was significantly higher in the winter than the summer months (0.013 vs −0.001 mm/month) [42].

**Studies with measurements at the level of the individual child**

Children with myopia spend less time outdoors than those without myopia, the difference being more marked on weekend days, as shown in a study with children wearing HOBO Pendants [21].

The cluster RCT in China testing 11 h or more of time outdoors reported reduced prevalence of new myopia onset and reduced progression in the intervention group: 14.47% vs 17.40% new onset myopia, 0.28 mm vs 0.33 mm mean axial elongation, 0.35F vs 0.47D myopic shift over 12 months [17].

The RCT exploring sending parents text messages twice daily for one year to remind them to take their children outdoors, axial elongation in SMS group was 0.27 mm (95%CI 0.24 to 0.30) at end of the intervention year, compared with 0.31 mm (95% CI 0.29 to 0.34; *P* = 0.03) in the control group. The spherical equivalent had increased by −0.42D (95% CI −0.34 to −0.50) in the SMS and by −0.51D (95% CI −0.43 to −0.59, *P* =) in the control group.

The impact persisted to the second and third year of the study, i.e. beyond the end of the SMS intervention at the end of year 1. At the end of year 3, axial elongation in SMS group was 0.30 mm (95%CI 0.27 to 0.33) vs 0.35 mm (95%CI 0.33 to 0.37, *P* = 0.005) in the control group. Spherical equivalent increase in year 3 was −0.47D (95% CI −0.54 to −0.39) in the SMS vs −0.60D (95% CI −0.67 to −0.53, *P* = 0.01) in the control group. [22] Myopia prevalence also differed significantly: 24.8% versus 28.6% at the end of the intervention year, and 46.6% vs 65.4% at the end of year 3 [22].

Reduced time outdoors and exposure to lower mean levels of logged illuminance as measured with an Actiwatch 2 was observed in children with myopia, who also had higher mean axial length [23]. Analysis of longitudinal data showed that over 12 months, axial length increased by 0.19 mm in children with myopia, compared with 0.05 mm in children without myopia [24]. In addition, over 12 months, low daily light exposure (mean illuminance 459 lx) was associated with higher axial elongation (0.13 mm) than moderate (842 lx, 0.06 mm) and high light exposure (1455 lx, 0.065 mm) [24].

The post-hoc analysis of ROAM data showed that children with myopia who spent more time in dim/mesopic light had more severe myopia [25].

The study exploring seasonal variation in outdoor light exposure observed that myopia parameters progressed faster in children with myopia than in the overall cohort, with an axial length increase of 0.41 mm vs 0.22 mm and spherical equivalent reduction of −0.44D vs 0.18D [29].

The study in Israel comparing different educational settings showed that schools with higher educational demands and less time outdoors had a higher prevalence of myopia amongst their students, although parental myopia was of similar prevalence at different schools. The number of studies acquiring measurements at the level of the individual child was greater than for those acquiring measurements at room or other surrounding levels, both for light-parameters (13 vs 4 studies) and for near-work parameters (5 vs 1, Table 3). Studies exploring light parameters were more frequent than those studying near-work parameters (17 vs 6 studies). Ultra-orthodox boys had a greater axial length (23.6 mm, SD 1.07) than religious (23.27 mm, SD 0.99) and secular boys (23.14 mm, SD 0.77) [28].

The study of light exposure and activity levels in the USA before and during the COVID-19 pandemic did not report the impact of different levels of light exposure on myopia parameter [27].

In the nested cohort from the GUSTO study in Singapore who provided individual light measurement data, measured light levels and duration of light exposure were not associated with prevalence of myopia, nor with axial length or spherical equivalent progression (*p* > 0.05), but diary-reported time outdoors was associated with lower odds of myopia (OR = 0.82, 95% CI 0.70 to 0.95/hour increase daily, *p* = 0.009) [33]. The authors recommend collecting both diary-reports as well as objective measurements, as the correlation between these can be poor [33].The observational study in China using the Mumu smartwatch reported that light exposure patterns with at least 15 min continuous time outdoors and no less than 2,000 lx were associated with a smaller myopic shift in refraction (−0.007D, 95% CI, −0.011 to −0.002D) [35].

In the study in China which prescribed an additional time outdoors, the 2-year cumulative incidence of myopia was 20.6% in the group that spent an additional 40 min a day outdoors, and 23.8% for those who spent an additional 80 min/day outdoors, and 24.9% in the control group [16]. The cumulative difference in incidence between control and the 40-min group was −4.3% (95% CI, −7.1% to −1.5%), and to the 80-min group −1.1% (95% CI, −4.1% to 1.9%) [16]. The 2-year change in SE was −0.84D (SD 0.77) and in AL, 0.55 mm (SD 0.33) in the 40-min group compare with SE change of −0.93D (SD 0.77) and AL change of 0.58 mm (SD 0.33) in the 80-min group and −0.98D (SD 0.76) and 0.61 mm (SD 0,33), respectively, in the control group, before adjustment [16].

In young people wearing violet-wavelength transmissible eyewear, axial elongation over 12 months was less than in those wearing standard lenses (substudy 1: violet-transmissible contact lenses 0,17 mm versus standard violet-blocking spectacle lenses 0.25 mm; substudy 2: violet-transmissible contact lenses 0.14 mm vs standard violet-blocking contact lenses 0.19 mm) [18].

#### Reports on near-work parameters only

##### Measures

Clouclip is spectacle-mounted device which logs working distance over time, from which time spent at defined distances can be calculated [48]. It incorporates a vibration system which was set to alert children when they used a working distance of less than 30 cm for more than 5 s; for the study, a control group received alerts when working distance fell below 60 cm for more than 45 min.

Kinect-for-Windows software can be used to measure the near-work environment; one study used this approach on children’s desk area at home [46]. The software captures a 3-dimensional image, whose depth values can be converted into “scene defocus” with respect to the child’s viewpoint and quantified as “dioptric volume”, the total amount of net defocus (DV).

In another app, a smart-phone based app was used to log working distance over time, providing mean daily duration of smartphone usage and face-to-screen distance [45].

##### Associations with and impact on myopia onset and progression

The dioptric volume (DV) at children’s desk at home, calculated using Kinect for Windows, and where they spent a median time of 2 h/day, with an average working distance of 29.7 cm (as estimated by parental questionnaires and measured with a tape-measure, respectively), was not correlated with myopia progression [46]. However, the regional DV at 15 to 20 degrees eccentricity did correlate with myopia progression [46].

In teenagers in the Netherlands, a mean face-to-screen distance of 29.1 cm has been observed using a custom-developed smartphone app, and a mean daily duration of smartphone screen viewing of 3.71 h on school days and 3.82 h on non-school days [45]. There was a mean 6.42 daily episodes of continuous smart-phone use on school days, and 7.10 on non-school days. Mean spherical equivalent and axial length in this study indicated that not all participants were myopic; a longitudinal study would be required to correlate smartphone use with myopia onset and progression.

#### Reports on both light and near-work parameters

##### Measures

Data logging of illuminance, providing simultaneous measurements of “light intensity” and exposure duration, as well as working distance and time spent at a defined working distance is possible at individual level with spectacle-mounted devices such as the Clouclip [30–32] and Akeso sensors [34], and these data can be combined into a derived parameter, the “visual behaviour index” [31].

##### Associations with and impact on myopia onset and progression

One study using Clouclip compared the “visual behaviour” of children with and without myopia, and observed that children with myopia spent less time per day in environments with illuminance > 3000 lx (0.68 h vs 1.02 h) and > 5000 lx (0.42 h vs 0.63 h). They also tended to spend more time on tasks with a working distance < 20 cm (1.89 h/day vs 1.52 h/day), and had a slightly lower habitual working distance (31.24 cm vs 33.86 cm) [30]. A second study reported differences between objective Clouclip measurements and questionnaire answers for average time per day spent on near work, maximum time for single episodes of near work, average near working distance, average total time spent on outdoor activities [32]. Logistic regression analysis shows that prolonged near work, shorter working distance and lesser outdoor activities were associated with myopia [32].

During the COVID-19 pandemic lockdowns, many educational activities were transferred to online platforms. This allowed comparisons of myopia progression during “online schooling” versus “on-site schooling”. Using the Akeso sensor, one study reported that axial elongation was higher during 6 months of online schooling than 6 months of on-site schooling; but this did not reach statistical significance. Spherical equivalent progressed significantly during online, but not during on-site schooling [34].

Analysis of the derived “visual behaviour index” indicates that as long as working distance is greater than 40 cm, near work may not promote myopia progression, regardless of the mean illuminance at eye-level throughout the day (“light intensity") [31]. Regardless of working distance, near-work did not appear to promote myopia progression as long as mean “light intensity” throughout the day was higher than 6300 lx [31]. These findings would support a message that a working distance of greater than 40 cm is important if daytime light exposures are low, and that increasing daytime light exposure reduces the impact of near-working distance.

#### Time outdoors as proxy for illuminance > 1000 lx

##### Measures

In a cluster-randomised trial in Taiwan, the recess outside the classroom (ROC) program, children to in the intervention-schools had to go outside for outdoor activities at school breaktimes [49]. The total daily recess time in school was 80 min (10, 20, and 10 min in both the morning and afternoon), and the total weekly recess time was approximately 6.7 h. Adherence was checked at classroom level, but no objective light exposure measurements were conducted [49].

In a similar study in China, the intervention group was allowed two additional compulsory 20-min recess programs outside the classroom per day over 12 months [50], and outdoor play equipment was provided to incentivise children to take part in activities. Again, light exposure was not measured objectively.

A longer trial running over 3 years introduced a mandatory additional 40-min outdoor activity class at the end of the school day during term-time [51]. In addition, engagement in outdoor activities outside school was promoted by giving parents and children special school bags, umbrellas, water bottles and hats with outdoor activity logos, and by providing a regular newsletter to parents. Incentivisation was used by rewarding children for completing a diary of weekend outdoor activities time [51]. Light exposure was not measured objectively.

In a non-randomised, school-based study in rural Beijing Districts, China, children in the intervention group took part in outdoor jogging for 30 min on every school day for a year; students were assessed before and for up to 3 years after the end of the intervention year [52]. Light exposure was not measured.

##### Associations with and impact on myopia onset and progression

After 12 months of implementing the “recess outside the classroom” programme, new cases of myopia were lower in the intervention than in the control schools (8.41% vs 17.5%) [49]. Mean myopic shift was also lower (−0.25D vs −0.38D) [49].

In the 12-month study in China where children spent an additional 40 min of school-breaktime outdoors per day, cases of new myopia were lower (3.70% vs 8.50%), myopic shift was lower (−0.10D vs −0.27D) and axial elongation was (0.16 mm vs 0.21 mm) [50].

In the 3-year study with an additional 40-min outdoor activity at the end of the school day showed a lower myopia incidence of 30.4% in the intervention schools, compared with 39.5% in the control schools [51]. Myopic shift and axial elongation were also lower (−1.42D vs −1.59D; 0.95 mm vs 0.98 mm) [51].

Children without myopia who took part in 30 min of outdoor jogging a day for a year, axial elongation and myopic shift of refraction were significantly lower than in a control group, but in children with myopia at baseline, there was no difference between intervention and control group [52].

## Discussion

### Summary of evidence

This scoping review presents a systematic overview of the literature on studies which used objective methods to measure exposures to light and near-work activities and their impact on myopia onset and progression. All included studies indicate that higher myopia prevalence and progression rates occur in children who spend less time in bright-light environments, more time in dim-light environments, and more time and sustained episodes longer than 30 min on near-vision activities, at shorter viewing distances, such as less than 20 or 30 cm [11]. The first intervention studies have emerged, increasing time outdoors or improving lighting in classrooms [39], alerting children to near-vision distance and duration [48].

### Research gaps

Most studies measured exposure to light at the level of the individual child. Given the increasing prevalence of myopia, population-based studies measuring exposures for groups of children, for example those studying in the same classrooms and sharing the same time of day outdoors, will extend the reach of research. One problem will be the variability of illuminance levels even within the same classroom [53]. However, whilst one methodological solution may be to exclude daylight, this would reduce illuminances and adversely affect the environment across the whole classroom.

As observed in other reviews, the regional distribution of study settings is clustered in East Asia [8]. The number of studies that used objective technologies and could be included in this review is small, but has grown since earlier systematic reviews [8, 10, 11, 13, 14]. Few studies have explored illuminance at eye-level in classrooms, outdoors at home or during leisure-activities, and few have reported selected near-work habits, such as smartphone screen viewing.

New techniques may offer the promise to deliver more accurate light exposure assessments at the level of a local group, i.e. a class of schoolchildren. These include the exploitation of satellite observational data relating to outdoor insolation [54] and architectural post-occupancy evaluation based on direct environmental measurements to create lighting performance models of classrooms and other indoor spaces regularly used by groups of children [55, 56] Existing satellite imagery models could be adapted to predict eye exposure profiles for different spectral regions as well as illuminance. Satellite data can also be used as input into architectural models to determine the effects of weather on indoor illuminance, compared to sky models used previously. Such methods might allow large-scale, unobtrusive and extended periods of light exposure assessments when wearable light loggers do not meet research objectives. Notably, these methods may be suited to capturing the impact of environmental interventions, such as additional school time outdoors and changes in classroom lighting designs.

### Limitations

In line with recommendations for scoping reviews, we did not appraise the quality of the included publications. The heterogeneity of included studies only allows a narrative overview. We also did not control for potential repeated analysis of individual participants or cohorts; as we included secondary analyses, sub-analyses of different data from the ROAM study were included here [23–25]. We included publications which reported light exposure without providing illuminance measurements [41, 49, 51]; however, time outdoors could be verified using meteorological data and school schedules. The measurement techniques adopted in included studies may also be suboptimal for the intended context. Ideally, illuminance logging devices should estimate the light arriving at the eye, and wearing the devices in other measurement positions, as well as obstructions from clothing, may have significant effects, particularly for devices worn at the wrist, and at higher illuminance levels. However, a measurement position at eye-level may feel more obtrusive than wrist or chest positions [15, 57].

## Conclusions

Included publications support an association between increases in incidence and progression of myopia and the environmental factors considered: reduced illuminance and/or less time spent in bright light, typically outdoor daylight; seasonal lows in daylight; and increased daily duration of near-vision activities and reduced working distance. Few studies have explored interventions targeting light exposure, lighting and near-vision activities, but those studies have demonstrated a protective impact on myopia parameters.

## Supplementary Information

Below is the link to the electronic supplementary material.

Supplementary file1 (DOCX 31 KB)
