# Supplementary material for: Measuring the visual environment of children and young people at risk of myopia: a scoping review
Source: Graefes Arch Clin Exp Ophthalmol. 2025 Jan 22;263(5):1213–28. doi: 10.1007/s00417-024-06719-z (PMC12148980; doi:10.1007/s00417-024-06719-z)
Supplement: Supplementary file 1 — Supplementary file1 (DOCX 31 KB) [file 417_2024_6719_MOESM1_ESM.docx]

Supplementary table 1. Search strategy Ovid MEDLINE 1946 to July 2022

| Search  lines | Search terms | Search results |
| --- | --- | --- |
| 1 | exp Myopia/ | 21111 |
| 2 | (Myopi* or Myopes or Short-sight* or Shortsight* or Short sight* or Near-sight* or Nearsight* or Near sight* or Refract* error or Refract* disorder* or Refract* shift* or Refract* chang* or Ocular refract* or refract* eye* or visual acuit* or vision acuit* or blur* vision or Ametropia).mp. [mp=title, book title, abstract, original title, name of substance word, subject heading word, floating sub-heading word, keyword heading word, organism supplementary concept word, protocol supplementary concept word, rare disease supplementary concept word, unique identifier, synonyms, population supplementary concept word, anatomy supplementary concept word] | 148198 |
| 3 | 1 or 2 | 148198 |
| 4 | exp Sunlight/ | 110298 |
| 5 | (sun or sunlight or sun light or daylight or day light or natural light).mp. | 61780 |
| 6 | exp Running/ | 23731 |
| 7 | exp Walking/ | 67296 |
| 8 | exp Football/ | 7605 |
| 9 | exp Soccer/ | 10201 |
| 10 | exp Rugby/ | 276 |
| 11 | exp Hockey/ | 1955 |
| 12 | exp Tennis/ | 1892 |
| 13 | exp Baseball/ | 2858 |
| 14 | exp Skating/ | 971 |
| 15 | exp Skiing/ | 3678 |
| 16 | (run or running or walk* or football or soccer or Rugby or Hockey or Tennis or Baseball or Cricket or Skating or Skiing or Cycling or Surfing or Sailing).mp. [mp=title, book title, abstract, original title, name of substance word, subject heading word, floating sub-heading word, keyword heading word, organism supplementary concept word, protocol supplementary concept word, rare disease supplementary concept word, unique identifier, synonyms, population supplementary concept word, anatomy supplementary concept word] | 446173 |
| 17 | (outdoor* or out-door* or open-air).mp. [mp=title, book title, abstract, original title, name of substance word, subject heading word, floating sub-heading word, keyword heading word, organism supplementary concept word, protocol supplementary concept word, rare disease supplementary concept word, unique identifier, synonyms, population supplementary concept word, anatomy supplementary concept word] | 34709 |
| 18 | exp Lighting/ | 12893 |
| 19 | exp Built Environment/ | 1366 |
| 20 | (built environment or illuminat*).mp. [mp=title, book title, abstract, original title, name of substance word, subject heading word, floating sub-heading word, keyword heading word, organism supplementary concept word, protocol supplementary concept word, rare disease supplementary concept word, unique identifier, synonyms, population supplementary concept word, anatomy supplementary concept word] | 84232 |
| 21 | (reduc* light or minimi* light or ((dim or dimmed or dimming) and light) or dusk or twilight or evening or night or nighttime or dark*).mp. | 230344 |
| 22 | 4 or 5 or 6 or 7 or 8 or 9 or 10 or 11 or 12 or 13 or 14 or 15 or 16 or 17 or 18 or 19 or 20 or 21 | 930212 |
| 23 | exp Computers/ | 86343 |
| 24 | exp Television/ | 32903 |
| 25 | exp Internet Access/ or exp Internet/ or exp Internet Addiction Disorder/ | 97696 |
| 26 | exp Electronics/ | 47634 |
| 27 | exp Computers, Handheld/ | 12974 |
| 28 | exp Mobile Applications/ or exp Cell Phone/ | 29840 |
| 29 | exp Screen Time/ | 1128 |
| 30 | exp Social Media/ | 15728 |
| 31 | exp Motion Pictures/ | 8438 |
| 32 | exp Wearable Electronic Devices/ or exp Smartphone/ | 27513 |
| 33 | exp Online Systems/ | 16678 |
| 34 | exp Virtual Reality/ | 5649 |
| 35 | exp Reading/ | 25652 |
| 36 | exp Writing/ | 41343 |
| 37 | (reading or writing or Computer* or tablet* or TV or televis* or internet or Electronic device* or ipad or iphone or mobile* or phone* or xbox or digital screen or screen or youtube or apps or app or movie or film or gadget or smartphone* or website or social media or facebook or twitter or Instagram or tiktok or blog* or video or ICT or information communication technology or zoom or online or virtual).mp. | 2195257 |
| 38 | 23 or 24 or 25 or 26 or 27 or 28 or 29 or 30 or 31 or 32 or 33 or 34 or 35 or 36 or 37 | 2272276 |
| 39 | 22 or 38 | 3103658 |
| 40 | 3 and 39 | 17157 |
| 41 | limit 40 to "all child (0 to 18 years)" | 4246 |

Supplementary table 2. Search strategy Ovid EMBASE 1947 to July 2022

| Search  lines | Search terms | Search results |
| --- | --- | --- |
| 1 | exp myopia/ | 34363 |
| 2 | (Myopi* or Myopes or Short-sight* or Shortsight* or Short sight* or Near-sight* or Nearsight* or Near sight* or Refract* error or Refract* disorder* or Refract* shift* or Refract* chang* or Ocular refract* or refract* eye* or visual acuit* or vision acuit* or blur* vision or Ametropia).mp. | 246125 |
| 3 | 1 or 2 | 246125 |
| 4 | exp sunlight/ | 23925 |
| 5 | (sun or sunlight or sun light or daylight or day light or natural light).mp | 94307 |
| 6 | exp running/ | 41859 |
| 7 | exp walking/ | 148853 |
| 8 | exp football/ | 13646 |
| 9 | exp soccer/ | 7958 |
| 10 | exp rugby/ | 3278 |
| 11 | exp hockey/ | 1542 |
| 12 | exp tennis/ | 4051 |
| 13 | exp baseball/ | 4221 |
| 14 | exp skating/ | 543 |
| 15 | exp skiing/ | 5945 |
| 16 | exp cycling/ | 16474 |
| 17 | (run or running or walk* or football or soccer or Rugby or Hockey or Tennis or Baseball or Cricket or Skating or Skiing or Cycling or Surfing or Sailing).mp | 670365 |
| 18 | (outdoor* or out-door* or open-air).mp | 45395 |
| 19 | exp illumination/ | 44175 |
| 20 | exp built environment/ | 2134 |
| 21 | (built environment or illuminat*).mp | 112662 |
| 22 | (reduc* light or minimi* light or ((dim or dimmed or dimming) and light) or dusk or twilight or evening or night or nighttime or dark*).mp | 351139 |
| 23 | 4 or 5 or 6 or 7 or 8 or 9 or 10 or 11 or 12 or 13 or 14 or 15 or 16 or 17 or 18 or 19 or 20 or 21 or 22 | 1260938 |
| 24 | exp computer/ | 188771 |
| 25 | exp computer/ | 188771 |
| 26 | exp internet access/ or exp Internet/ or exp internet addiction/ | 138344 |
| 27 | exp electronics/ | 88327 |
| 28 | exp tablet computer/ | 3728 |
| 29 | exp mobile phone addiction/ or exp mobile phone/ | 51443 |
| 30 | exp "cell phone use"/ or exp mobile phone addiction/ or exp mobile phone/ | 52539 |
| 31 | exp social media/ | 53806 |
| 32 | exp movie/ | 3484 |
| 33 | exp mobile phone/ or exp smartphone/ or exp microcomputer/ | 65175 |
| 34 | exp screen time/ | 3307 |
| 35 | exp virtual reality/ | 28497 |
| 36 | exp reading | 64289 |
| 37 | exp writing/ | 49927 |
| 38 | (reading or writing or Computer* or tablet* or TV or televis* or internet or Electronic device* or ipad or iphone or mobile* or phone* or xbox or digital screen or screen or youtube or apps or app or movie or film or gadget or smartphone* or website or social media or facebook or twitter or Instagram or tiktok or blog* or video or ICT or information communication technology or zoom or online or virtual).mp | 3796081 |
| 39 | 24 or 25 or 26 or 27 or 28 or 29 or 30 or 31 or 32 or 33 or 34 or 35 or 36 or 37 or 38 | 3867996 |
| 40 | 23 or 39 | 4964482 |
| 41 | 3 and 40 | 39596 |
| 42 | limit 41 to (infant <to one year> or child <unspecified age> or preschool child <1 to 6 years> or school child <7 to 12 years> or adolescent <13 to 17 years>) | 7260 |

Supplementary table 3. The Cochrane Central Register of Controlled Trials

| Search  lines | Search terms | Search results |
| --- | --- | --- |
| 1 | MeSH descriptor: [Myopia] explode all trees | 1660 |
| 2 | Myopi* or Myopes or Short-sight* or Shortsight* or Short sight* or Nearsight* or Refract* error or Refract* disorder* or Refract* shift* or Refract* chang* or Ocular refract* or refract* eye* or visual acuit* or vision acuit* or blur* vision or Ametropia | 31594 |
| 3 | #1 or #2 | 31594 |
| 4 | MeSH descriptor: [Sunlight] explode all trees | 1601 |
| 5 | sun or sunlight or sun light or daylight or day light or natural light | 27749 |
| 6 | MeSH descriptor: [Running] explode all trees | 2749 |
| 7 | MeSH descriptor: [Walking] explode all trees | 8200 |
| 8 | MeSH descriptor: [Football] explode all trees | 398 |
| 9 | MeSH descriptor: [Rugby] explode all trees | 30 |
| 10 | MeSH descriptor: [Hockey] explode all trees | 95 |
| 11 | MeSH descriptor: [Tennis] explode all trees | 112 |
| 12 | MeSH descriptor: [Baseball] explode all trees | 105 |
| 13 | MeSH descriptor: [Skating] explode all trees | 29 |
| 14 | MeSH descriptor: [Skiing] explode all trees | 162 |
| 15 | run or running or walk* or football or soccer or Rugby or Hockey or Tennis or Baseball or Cricket or Skating or Skiing or Cycling or Surfing or Sailing | 88389 |
| 16 | outdoor* or out-door* or open-air | 2141 |
| 17 | MeSH descriptor: [Lighting] explode all trees | 308 |
| 18 | MeSH descriptor: [Built Environment] explode all trees | 26 |
| 19 | built environment or illuminat* | 2402 |
| 20 | (reduc* light or minimi* light or ((dim or dimmed or dimming) and light) or dusk or twilight or evening or night or nighttime or dark*) | 2130732 |
| 21 | #4 or #5 or #6 or #7 or #8 or #9 or #10 or #11 or #12 or #13 or #14 or #15 or #16 or #17 or #18 or #19 or #20 | 2130964 |
| 22 | MeSH descriptor: [Computers] explode all trees | 2989 |
| 23 | MeSH descriptor: [Television] explode all trees | 1776 |
| 24 | MeSH descriptor: [Internet] explode all trees | 6494 |
| 25 | MeSH descriptor: [Electronics, Medical] explode all trees | 97 |
| 26 | MeSH descriptor: [Computers, Handheld] explode all trees | 1539 |
| 27 | MeSH descriptor: [Cell Phone] explode all trees | 3449 |
| 28 | MeSH descriptor: [Smartphone] explode all trees | 1141 |
| 29 | MeSH descriptor: [Social Media] explode all trees | 567 |
| 30 | MeSH descriptor: [Motion Pictures] explode all trees | 222 |
| 31 | MeSH descriptor: [Screen Time] explode all trees | 77 |
| 32 | MeSH descriptor: [Virtual Reality] explode all trees | 1080 |
| 33 | MeSH descriptor: [Reading] explode all trees | 1216 |
| 34 | MeSH descriptor: [Writing] explode all trees | 3074 |
| 35 | (reading or writing or Computer* or tablet* or TV or televis* or internet or Electronic device* or ipad or iphone or mobile* or phone* or xbox or digital screen or screen or youtube or apps or app or movie or film or gadget or smartphone* or website or social media or facebook or twitter or Instagram or tiktok or blog* or video or ICT or information communication technology or zoom or online or virtual) | 272497 |
| 36 | #22 or #23 or #24 or #25 or #26 or #27 or #28 or #29 or #30 or #31 or #32 or #33 or #34 or #35 | 274197 |
| 37 | #21 or 36 | 2132528 |
| 38 | #3 and #37 | 31540 |
| 39 | MeSH descriptor: [Youth Sports] explode all trees | 29 |
| 40 | MeSH descriptor: [Child] explode all trees | 81477 |
| 41 | child* or p?ediatric* or adolescen* | 352864 |
| 42 | #39 or #40 or #41 | 352964 |
| 43 | #38 and #42 | 4904 |
